# A Reliable Method for Rhythm Analysis during Cardiopulmonary Resuscitation

**DOI:** 10.1155/2014/872470

**Published:** 2014-05-07

**Authors:** U. Ayala, U. Irusta, J. Ruiz, T. Eftestøl, J. Kramer-Johansen, F. Alonso-Atienza, E. Alonso, D. González-Otero

**Affiliations:** ^1^Communications Engineering Department, University of the Basque Country UPV/EHU, Alameda Urquijo S/N, 48013 Bilbao, Spain; ^2^Department of Electrical Engineering and Computer Science, Faculty of Science and Technology, University of Stavanger, 4036 Stavanger, Norway; ^3^Norwegian Centre for Prehospital Emergency Care (NAKOS), Oslo University Hospital and University of Oslo, 0424 Oslo, Norway; ^4^Department of Signal Theory and Communications, University Rey Juan Carlos, Camino del Molino S/N, 28943 Madrid, Spain

## Abstract

Interruptions in cardiopulmonary resuscitation (CPR) compromise defibrillation success. However, CPR must be interrupted to analyze the rhythm because although current methods for rhythm analysis during CPR have high sensitivity for shockable rhythms, the specificity for nonshockable rhythms is still too low. This paper introduces a new approach to rhythm analysis during CPR that combines two strategies: a state-of-the-art CPR artifact suppression filter and a shock advice algorithm (SAA) designed to optimally classify the filtered signal. Emphasis is on designing an algorithm with high specificity. The SAA includes a detector for low electrical activity rhythms to increase the specificity, and a shock/no-shock decision algorithm based on a support vector machine classifier using slope and frequency features. For this study, 1185 shockable and 6482 nonshockable 9-s segments corrupted by CPR artifacts were obtained from 247 patients suffering out-of-hospital cardiac arrest. The segments were split into a training and a test set. For the test set, the sensitivity and specificity for rhythm analysis during CPR were 91.0% and 96.6%, respectively. This new approach shows an important increase in specificity without compromising the sensitivity when compared to previous studies.

## 1. Introduction

Out-of-hospital cardiac arrest (OHCA) is a leading cause of mortality in the industrialized world, with an estimated annual incidence between 28 and 55 cases per 100,000 person-years [1]. Early cardiopulmonary resuscitation (CPR) and early defibrillation are the key interventions for survival after cardiac arrest [2]. Defibrillation may be administered by an automated external defibrillator (AED), which incorporates a shock advice algorithm (SAA) that analyzes the ECG to detect shockable rhythms. Current CPR guidelines emphasize the importance of high quality CPR with minimal interruptions in chest compressions (CCs) [3]. However, CPR must be interrupted for a reliable rhythm analysis because CCs produce artifacts in the ECG. These interruptions adversely affect the probability of defibrillation success and subsequent survival [4]. Currently, CPR is interrupted every 2 minutes for rhythm reassessment on an artifact-free ECG.

Although different approaches to rhythm analysis during CPR have been explored, for instance, algorithms that directly diagnose the ECG corrupt with CPR artifacts [5, 6], filtering the CPR artifact has been a major approach (see [7] for a comprehensive review). The time-varying characteristics of the CPR artifact and its spectral overlap with both shockable and nonshockable cardiac arrest rhythms mandate the use of adaptive filters [8], which use reference signals to model the CPR artifact. Over the years, many solutions have been proposed, including Wiener filters [9], Matching Pursuit Algorithms [10], Recursive Least Squares [11], least mean squares (LMS) [12], or Kalman filters [13, 14]. Adaptive solutions using exclusively the ECG have also been explored [15, 16], but the results were poorer. To evaluate the performance of these methods, researchers first filtered the CPR artifact and then analyzed the rhythm using a SAA to obtain the sensitivity and specificity of the method, that is, the proportion of correctly diagnosed shockable and nonshockable rhythms, respectively. However, the SAAs used were originally designed to analyze artifact-free ECG instead of the ECG after filtering.

Currently rhythm analysis during CPR is not possible [17]. Most methods have sensitivity above 90%, the minimum value recommended by the American Heart Association (AHA) for SAA on artifact-free ECG [18]. However, specificity rarely exceeds 85%, well below the 95% value recommended by the AHA. A low specificity would result in a large number of false shock diagnoses during CPR, which would unnecessarily increase the number of interruptions in CPR. Overall, the main cause of the low specificity is filtering residuals in nonshockable rhythms. These residuals frequently resemble a disorganized rhythm [10, 12] and are often misdiagnosed as shockable by SAAs designed to analyze artifact-free ECG. This problem is more prominent when the electrical activity of the underlying heart rhythm is low, particularly for asystole (ASY) [14, 16], because filtering residuals may have amplitudes comparable or larger than those of the underlying ECG.

In this study we explore the possibility of combining adaptive filtering techniques with a SAA designed to optimally classify the rhythm after filtering. The aim is to improve the accuracy of current approaches and in particular to overcome the low specificity. When compared to previous studies, our results showed an increased specificity without compromising the sensitivity, for a comprehensive dataset of OHCA rhythms.

## 2. Materials and Methods

### 2.1. Data Collection

The data for this study were extracted from a large prospective study of OHCA conducted between 2002 and 2004 in three European sites [21, 22]. CPR was delivered by trained ambulance personnel in adherence to the 2000 resuscitation guidelines. Episodes were recorded using modified Laerdal Heartstart 4000 defibrillators (4000SP) and an external CPR assist pad to acquire additional reference signals. All signals were acquired with a 500 Hz sampling rate. The initial rhythm and all subsequent changes in rhythm were annotated by consensus of an experienced anesthesiologist and a biomedical engineer, both specialized in resuscitation [21, 22]. Rhythm annotations comprised five types (see [21] for further details): VF and fast ventricular tachycardia (VT) in the shockable category and ASY, pulseless electrical activity (PEA), and pulse-generating rhythm (PR) in the nonshockable category. Intervals with chest compressions were annotated using the compression depth (CD) obtained from the CPR assist pad.

For this study specific records containing the ECG and CD signals were automatically extracted from the original episodes. First rhythm transitions were identified using the original annotations, and then for each interval without rhythm transitions at most one record was extracted to avoid bias due to data selection. Records were extracted if the following criteria were met: duration of more than 20-s, ongoing CCs, and the same rhythm annotation before and after CCs. Following the AHA statement the records were grouped into a shockable and a nonshockable category. The amplitude thresholds adopted for coarse VF and ASY are those accepted in the literature on SAAs [6, 18]. The following criteria and rhythm definitions were checked in the clean intervals before and after CCs.

*Shockable Rhythms.* This category includes fast VT, with rate above 150 beats per minute (bpm), and coarse VF. Coarse VF was defined as VF with peak-to-peak amplitude above 200 *μ*V and a fibrillation frequency above 2 Hz.

*Nonshockable Rhythms.* These rhythms were further divided into the following:

- organized rhythms (ORG): all nonshockable rhythms except ASY (PEA and PR),
- asystole (ASY): rhythms with peak-to-peak amplitudes below 100 *μ*V for at least 2-s.

All signals were resampled to *f*_*s*_ = 250 Hz, a sampling rate similar to that used by commercial AEDs. In what follows, the sample index and time variables are related by *t* = *n* · *T*_*s*_  (*T*_*s*_ = 1/*f*_*s*_). The ECG was band limited to 0.5–30 Hz (order 10 Butterworth filter), a typical ECG monitor bandwidth used in AEDs [5, 6], which removes base line wander and high frequency noise.

Following standard practice in SAA design, the rhythm analysis method was designed to analyze three consecutive 3 s windows, so it gives a diagnosis every 9 s [23, 24]. A 3 s window is sufficient to characterize the rhythm in terms of rate, stability, and morphology and to make a shock (Sh) or no-shock (NSh) decision [23]. SAA algorithms combine several consecutive diagnoses to avoid errors due to rhythm transitions and to avoid shock diagnoses for short bursts of nonsustained VT. Therefore, each record was divided into nonoverlapping 9 s segments. The 9 s segments were randomly split into two separate sets, one to train the algorithm and an independent set to test the algorithm, as required by the AHA statement. In addition we made sure that the patients on both sets were different (AHA statement) and that the distribution of rhythm types was similar in both sets.

### 2.2. Rhythm Analysis Method

**Figure 1:**
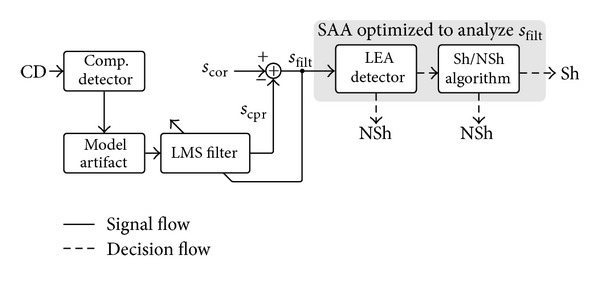
Block diagram of the new approach to rhythm analysis during CPR in which an adaptive filter (LMS filter based on the CD signal) is used in combination with a SAA designed to optimally classify the filtered signal, *s*_filt_.

The block diagram of the rhythm analysis method is shown in Figure 1. First, a CPR artifact suppression filter estimates the underlying rhythm, that is, the filtered ECG signal, *s*_filt_. Then, a SAA diagnoses every 3 s window of the filtered signal. The SAA is designed to optimally classify the filtered signal and is further composed of two sequential subalgorithms: (1) a detector of rhythms with low electrical activity (LEA), that is, nonshockable rhythms without distinguishable QRS complexes such as ASY or idioventricular rhythms, and (2) a Sh/NSh algorithm that classifies windows with electrical activity as shockable or nonshockable.

### 2.3. Chest Compression Artifact Filter

**Figure 2:**
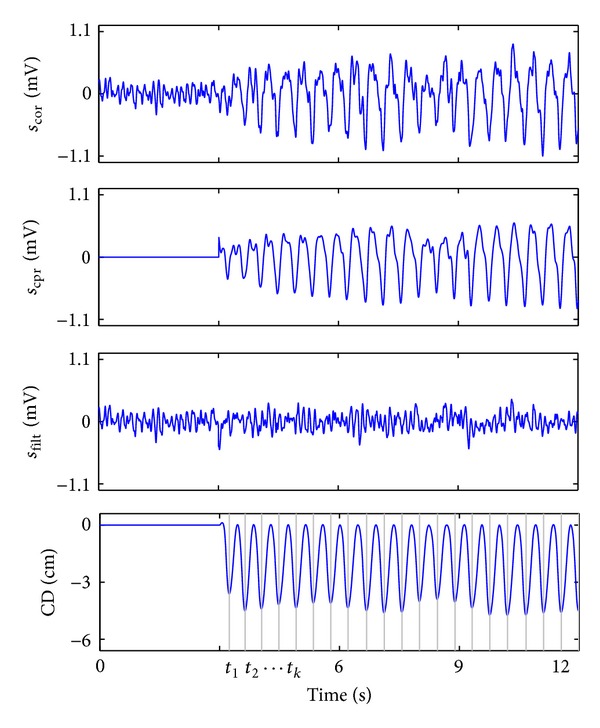
Filtering example of a 12-s segment. In the first 3 s there is no artifact and the underlying VF is visible. The filter estimates the artifact, *s*_cpr_, using information derived from the CC marks, indicated by vertical lines in CD (*t*_*k*_ instants).

CPR artifacts were suppressed using a state-of-the-art method based on an LMS filter [12]. In this method, CC artifacts are modeled as a quasiperiodic interference with a time-varying fundamental frequency, *f*_*o*_(*n*), which is the instantaneous frequency of the CCs. This frequency is derived from the *t*_*k*_ instants, the CC marks shown in Figure 2. The LMS algorithm adaptively estimates the time-varying amplitudes, *c*_*k*_(*n*), and phases, *ϕ*_*k*_(*n*), of the first 5 harmonics of the artifact by fitting the following model:

(1) scpr(n)=∑k=15ck(n)cos⁡(k·2πfo(n)·n+ϕk(n)),fo(n)=1tk−tk−1 for  tk−1≤nTs<tk.

In summary, the LMS algorithm dynamically estimates the CPR artifact by adaptively estimating its harmonic content. For this study, we used the optimal values of the filter parameters as described in [12, 19]. As shown in Figure 1, the filtered signal was obtained by subtracting the estimated CPR artifact from the corrupted ECG. Figure 2 shows those signals for a 12-s segment with an underlying VF rhythm.

### 2.4. Shock Advice Algorithm

The SAA consists of a LEA detector followed by the Sh/NSh algorithm. The LEA detector identifies LEA windows as nonshockable; the rest of the windows are further processed by the Sh/NSh algorithm for a definitive diagnosis.

#### 2.4.1. LEA Rhythm Detector

**Figure 3:**
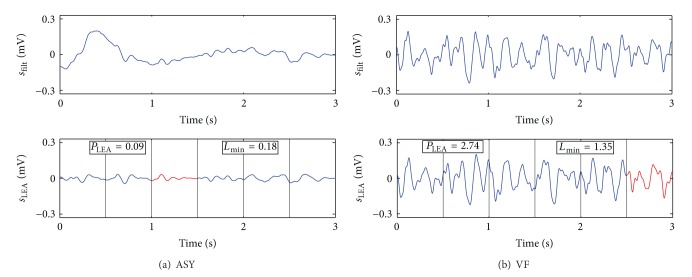
Examples of a LEA rhythm (a) and a VF (b) window after filtering the CC artifact, *s*_filt_, and preprocessed to suppress components under 2.5 Hz, *s*_LEA_. Vertical lines separate the 0.5 subintervals, and the one with lowest activity (*L*_min⁡_) is shown in red.

Some nonshockable rhythms (ASY, bradyarrhythmias or idioventricular rhythms) may not present QRS complexes in a 3 s analysis window. In these cases, filtering the CC artifact results in *s*_filt_ with low amplitudes and short intervals in which the electrical activity is very low (see Figure 3(a)). To further improve LEA detection *s*_filt_ was high pass filtered with a 2.5 Hz cut-off frequency using an order 5 Butterworth filter, which removed slow fluctuations of the ECG in LEA rhythms but preserved most frequency components of VF, as shown in Figure 3. The resulting signal, *s*_LEA_, was used to obtain the following two features:

(i) *P*_LEA_: energy of *s*_LEA_ in the 3 s window:
  (2) $$\begin{array}{c} P_{\text{LEA}}=\underset{n}{\sum }s_{\text{LEA}}^{2}\left(n\right); \end{array}$$
(ii) *L*_min⁡_: minimum of the curve lengths of *s*_LEA_ for nonoverlapping 0.5-s intervals, which measures the minimum electrical activity in 0.5-s intervals. In discrete form, the curve length of the *k*th subinterval is [25]
  (3) $$\begin{array}{c} L_{k}=\overset{(k+1)f_{s}/2}{\underset{n=kf_{s}/2+1}{\sum }}\sqrt{\Delta s_{\text{LEA}}^{2}\left(n\right)+T_{s}^{2}}, \end{array}$$
 where Δ*s*_LEA_ is the first difference of *s*_LEA_.

LEA rhythms have smaller values of *P*_LEA_ and *L*_min⁡_ than shockable rhythms, as shown in Figure 3. This block was designed as a detector; that is, it gives a NSh diagnosis if a LEA rhythm is detected; otherwise the window is further processed by the Sh/NSh algorithm.

#### 2.4.2. Sh/NSh Algorithm

During resuscitation, ORG rhythms with electrical activity may be very different in terms of rate, QRS width, or QRS morphology. Furthermore, even after CPR artifact suppression, rhythms may present important filtering residuals that may resemble VF. Four features derived from the frequency domain and slope analyses were defined. For rhythms with electrical activity, these features emphasize the differences between nonshockable (with QRS complexes) and shockable (without QRS complexes) rhythms.

#### 2.4.3. Slope Analysis Features

**Figure 4:**
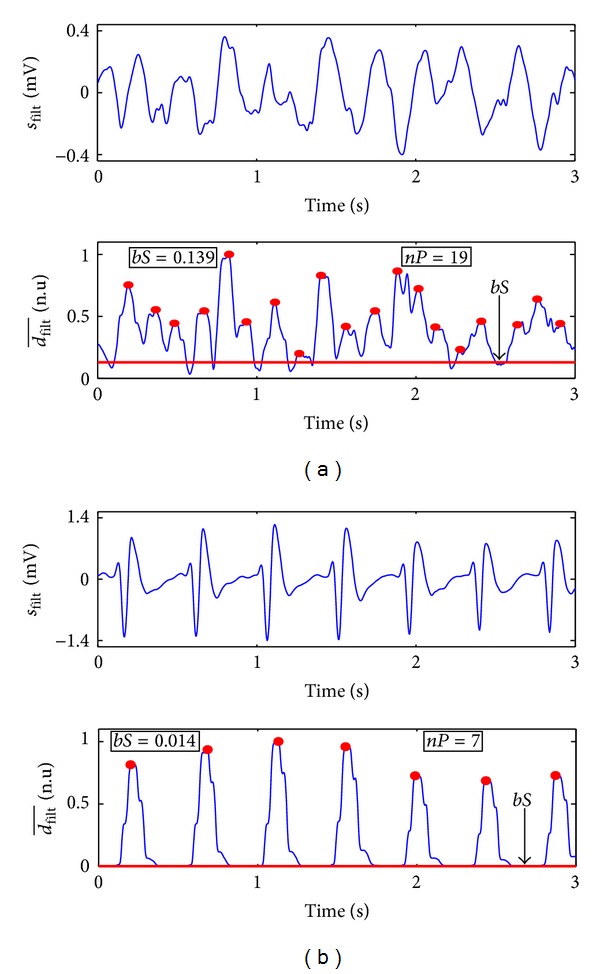
Example of the slope analysis for VF (a) and an ORG (b) window. During VF the slope, $\bar{d_{\text{filt}}}$, is irregular with many peaks, whereas ORG rhythms are regular with fewer peaks and concentrate most $\bar{d_{\text{filt}}}$ values around the baseline.

QRS complexes were enhanced in *s*_filt_ by computing the moving average of the square of its first difference (its slope):

(4) $$\begin{array}{c} d_{\text{filt}}\left(n\right)=\frac{1}{N}\overset{N-1}{\underset{k=0}{\sum }}{\left(s_{\text{filt}}(n-k)-s_{\text{filt}}\left(n-k-1\right)\right)}^{2}, \end{array}$$

where *N* corresponds to the number of samples in a 100 ms interval. Then, *d*_filt_ was divided by its maximum value in the analysis window to obtain $\bar{d_{\text{filt}}}$. As shown in Figure 4, in ORG rhythms $\bar{d_{\text{filt}}}$ is large only around QRS complexes and very small otherwise, whereas in VF the values of $\bar{d_{\text{filt}}}$ are more evenly distributed and present many peaks. Two features were defined to measure these differences:

- *bS*: slope baseline, a measure of how concentrated slope values are around small values (baseline), computed as the 10th percentile of $\bar{d_{\text{filt}}}$,
- *nP*: number of peaks above a fixed threshold in $\bar{d_{\text{filt}}}$.

Shockable rhythms will present larger values of *bS* and *nP* as shown in Figure 4.

#### 2.4.4. Frequency Domain Features

**Figure 5:**
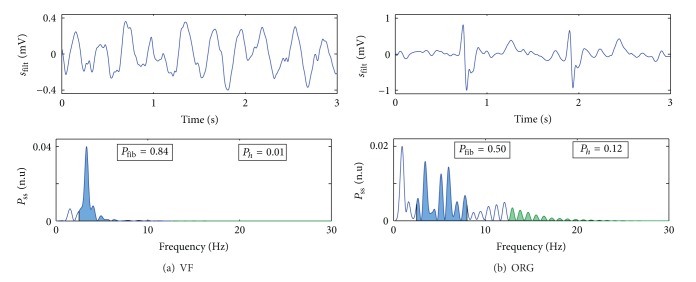
Example of the frequency domain analysis for VF (a) and an ORG (b) window. VF concentrates most of its power around the fibrillation band (blue). ORG rhythms have a spectrum with many harmonics of the heart rate and thus larger *P*_*h*_ (in green).

For the frequency analysis, a Hamming window was applied to *s*_filt_ and its zero padded 1024-point FFT was computed. The power spectral density was estimated as the square of the magnitude of the FFT and normalized to total power of one to give *P*_ss_(*f*). As shown in Figure 5, VF concentrates most of its power around the fibrillation frequency, whereas ORG rhythms may have important power content at higher frequencies, on the harmonics of the heart rate. Two discrimination features were defined, with limiting frequencies in line with the characteristics of human VF [26, 27]:

- *P*_fib_: power proportion around the VF-fibrillation band (2.5–7.5 Hz),
- *P*_*h*_: power proportion in the high spectral bands (above 12 Hz).

Shockable rhythms have larger values of *P*_fib_ but lower values of *P*_*h*_ (see Figure 5).

#### 2.4.5. Support Vector Machine (SVM) Classifier

The Sh/NSh algorithm classified windows using a SVM with a Gaussian kernel [28]. First, features were standardized to zero mean and unit variance using the data in the training set. These **x**_*i*_ vectors of four normalized features were arranged as {(**x**_1_, *y*_1_),…, (**x**_*n*_, *y*_*n*_)} ∈ *R* ^4^ × {±1}, where *y*_*i*_ = 1 for shockable and *y*_*i*_ = −1 for nonshockable windows. After training, the discriminant function for a window with feature vector **x** is

(5) $$\begin{array}{c} f\left(x\right)=\overset{N_{s}}{\underset{i=1}{\sum }}\alpha_{i}y_{i}\exp\left(-\gamma {||x-x_{i}||}^{2}\right)+b, \end{array}$$

where **x**_*i*_ are the support vectors, *N*_*s*_ is the number of support vectors, and *α*_*i*_ and *b* are coefficients estimated during training. Windows were classified as shockable for *f*(**x**) > 0 or nonshockable for *f*(**x**) ≤ 0. Selecting an optimal SVM model for the classification problem involves selecting two parameters: *C* and *γ*. The width of the Gaussian kernel, *γ*, determines the flexibility of the decision boundary [28]. The soft margin parameter, *C*, is used exclusively in the optimization process and is a tradeoff between classification errors in training data and separating the rest of the training data with maximum margin [28].

### 2.5. Data Analysis and Algorithm Optimization

The rate and depth characteristics of CPR in our data were analyzed for each 9 s segment. The distributions for rate and depth did not pass the Kolmogorov-Smirnov test for normality and are reported as median and 5–95 percentiles.

For each discrimination feature of the SAA, statistical differences in medians between the targeted classification groups of each subalgorithm were measured using the Mann-Whitney *U* test. The optimization process was carried out for the 3 s windows of the training set in two sequential steps.

(1) * LEA Detector*. ASY and shockable rhythms were used. The detection thresholds were determined through a greedy search on the two-dimensional feature space to jointly maximize the number of detected ASY and minimize the number of shockable windows incorrectly detected as nonshockable. An additional restriction was imposed: at maximum 5% of shockable windows could be incorrectly classified.
(2) * Sh/NSh Algorithm*. Shockable and ORG windows not detected as NSh by the LEA detector were used to optimize the SVM classifier. To avoid overfitting the SVM to the training set, *C* and *γ* were selected using 5-fold crossvalidation [29] to optimize the balanced error rate (BER):
  (6) $$\begin{array}{c} \text{BER}=1-\frac{1}{2}\left(\text{TPR}+\text{TNR}\right), \end{array}$$
 where the true positive rate (TPR) and the true negative rate (TNR) are the capacity of the SVM classifier to detect shockable and ORG windows, respectively. Weights were assigned to each class to resolve the unbalance in the number of instances per class [28]. The best SVMs using one, two, or three features were compared to the optimal four-feature SVM using McNemar's test.

The performance of the algorithm was measured in the test set in terms of sensitivity and specificity. Since both 3 s windows and 9 s segments correspond to consecutive analyses within a record, the sensitivities, specificities, and their 90% low one-sided confidence intervals (CI) were adjusted for clustering (longitudinal data) within each record, using a longitudinal logistic model fit by generalized estimating equations (GEE) [30, 31]. The analysis was carried out in *R* using the geepack library [32]. Finally, the algorithm was programmed in MATLAB R2013a (Mathworks Inc.) for Windows and processing time performance tests were carried out on a 2.9 GHz Intel i7 with 4 GB of RAM.

## 3. Results

### 3.1. Database Description

Our data comprise 7667 9 s segments within 1396 records extracted from 247 OHCA patient episodes. The median number of 9 s segments per record was 3 (1–19, range 1–44).  Table 1 shows the number of 9 s segments and the rate and depth of CCs for those segments in the training and test sets. The median CC rate and depth were 116 (88–156) compressions per minute (cpm) and 36 (21–53) mm, respectively.

### 3.2. Shock Advice Algorithm

#### 3.2.1. Training

**Figure 6:**
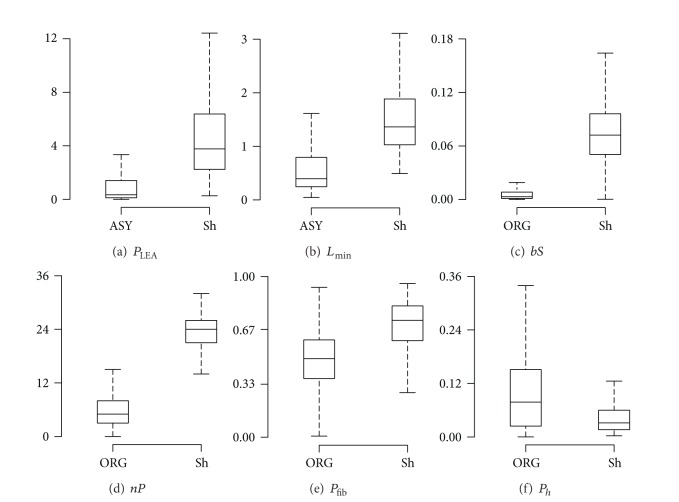
Features of the SAA for the rhythm types used in each training stage of the algorithm. For the LEA detector the figure compares ASY versus Sh (panels (a) and (b)), and for the SVM classifier ORG versus Sh (panels (c)–(f)). The boxes show the median and interquartile ranges (IQR) and the whisker shows the last datum within the ±1.5IQR interval. Significant differences were found for the median value of the features between the targeted groups (*P* < 0.0001).

Figures 6(a) and 6(b) show the values of *P*_LEA_ and *L*_min⁡_ for the ASY and shockable rhythms which presented significant differences between the two groups (*P* < 0.001). The optimal detection thresholds of the LEA detector were

(7) $$\begin{array}{c} P_{\text{LEA}}<0.44\;\text{or}\;L_{\min}<0.63. \end{array}$$

The LEA detector identified as NSh 72.1% of the ASY (true detections) and 0.9% of the shockable (false detections) windows. In addition, 38.8% of the ORG windows were correctly identified as NSh; these rhythms corresponded to very low rate and low electrical activity intervals of ORG rhythms.

Figures 6(c)–6(f) show the values of the features used in the SVM classifier; these values were statistically different for the ORG and shockable rhythms (*P* < 0.001). The SVM based on four features showed a significantly better performance when compared to the SVMs based on the best single, pair, or triplet of features (McNemar's test *χ* ^2^ > 10, *P* < 0.001, in all three cases). The optimal working point of the four-feature SVM was (*C* = 8.5, *γ* = 0.1), which produced a BER = 0.064, TPR = 0.927, and TNR = 0.944 for the SVM classifier. The receiver operating characteristics analysis on the SVM features resulted in the following area under the curve (AUC) values: 0.948, 0.928, 0.807, and 0.733 for *bS*, *nP*, *P*_fib_, and *P*_*h*_, respectively. When combined in the SVM the resulting AUC was 0.971, which reveals the robustness of the classifier.

#### 3.2.2. Test

The optimized SAA was used to classify the 3 s windows in the test set; Table 2 shows a summary of the results. The overall sensitivity and specificity were 89.7% (low one-sided 90% CI, 85.5) and 95.1% (low 90% CI, 94.3), respectively. The 9 s segments were diagnosed using a majority criterion on three consecutive window analyses, this increased the overall sensitivity and specificity to 91.0% (low 90% CI, 86.6) and 96.6% (low 90% CI, 95.9), respectively, and AHA recommendations were met for all rhythm types (see Table 2).

**Figure 7:**
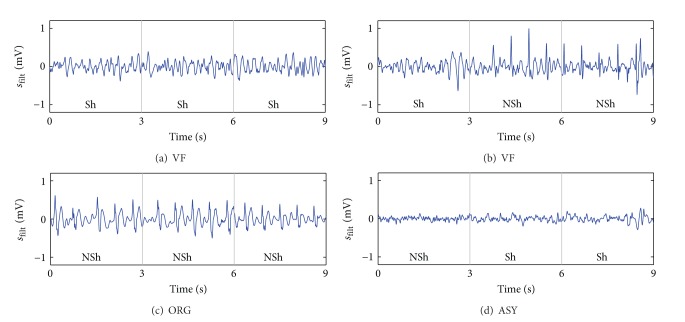
Examples of correctly and incorrectly classified 9 s segments. Examples (a, c) are correctly classified despite the presence of large filtering residuals. However, in the VF of panel (b) spiky filtering artifacts cause the erroneous classification. In the ASY of panel (d) filtering residuals are large in the last two windows causing the shock diagnosis.

Figure 7 shows two examples (Figures 7(a) and 7(c)) of correctly diagnosed segments and two examples (Figures 7(b) and 7(d)) of incorrectly diagnosed segments. The examples (Figures 7(a) and 7(c)) show that the algorithm works robustly even in the presence of important filtering residuals. However, there were some instances of misdiagnosed segments as shown in Figures 7(b) and 7(d). Errors were generally caused by spiky filtering residuals in shockable rhythms (Figure 7(b)) or large filtering residuals during ASY (Figure 7(d)).

Processing time for the complete algorithm, CPR suppression filter based on the LMS filter followed by the SAA, was on average 8.7 ms per 3 s segment. Processing time was broken down into 5.8 ms for the LMS filter and 2.9 ms for the SAA. For decisions taken by the LEA detector the SAA required only 1.8 ms, and for windows in which the LEA detector and the SVM were used it increased to 4.1 ms. In the worst case scenario processing time for the complete algorithm was under 10 ms.

## 4. Discussion

This study presents the first attempt to combine two approaches for rhythm analysis during CPR: adaptive filters to suppress the CPR artifact and an SAA optimized to analyze the rhythm after filtering. Our objective was to increase the specificity, because the low specificity of current methods has restrained their implementation in current defibrillators. Our results indicate that our new design approach might contribute to a substantial increase of the accuracy of rhythm analysis methods during CPR, with results that marginally meet AHA performance goals.

The design efforts were focused on obtaining a high specificity during CPR to allow CCs to continue uninterrupted until the method gives a shock advice. The positive predictive value (PPV) of the algorithm, that is, the confidence in a shock diagnosis, must be kept high to avoid unnecessary CPR interruptions if the underlying rhythm is nonshockable. Since VF is the positive class, the PPV depends on the sensitivity/specificity of the algorithm and on the prevalence of VF, *P*_vf_, in the following way:

(8) PPV(%)=100×TPTP+FP=100×Se·PvfSe·Pvf+(1−Sp)·(1−Pvf).

The exact prevalence of VF (reported for the initially observed rhythm as stated in [33]) is unknown and varies among OHCA studies, with figures in the range of 23% to 67% [34, 35]. For the original OHCA studies from which our datasets originated the prevalences of VF were 43% [21] and 41% [22], within the previous range. For the limits of the VF prevalence range, the PPV of our algorithm is high, in the 88.9% to 98.2% range. Furthermore, since the PPV depends on the prevalences, algorithms must be trained to optimize sensitivity/specificity, with emphasis on a large specificity (a specificity of 100% would result in a PPV of 100% regardless of the prevalences).

To this date most methods for rhythm analysis during CPR have focused on the accurate detection of shockable rhythms, resulting in higher values for sensitivity than for specificity.  Table 3 compares the accuracy of our method to that of five well-known methods tested on OHCA data that represent the two most successful strategies for rhythm analysis during CPR. Three of those methods are based on adaptive filters [10, 12, 20], and the other two are algorithms designed to directly diagnose the corrupt ECG [5, 6]. Although the sensitivity of our method is up to 4 points below that reported by methods based on adaptive filters, it is still above the value recommended by the AHA, which ensures the detection of a high proportion of shockable rhythms. The higher sensitivity of methods based on adaptive filters may be explained by the fact that filtering residuals are frequently diagnosed as shockable by SAA designed to diagnose artifact-free ECG [14]. In contrast, the 96.6% specificity of our approach is an important improvement with respect to previous approaches in which the specificity was below 91%. We showed that combining the strong points of both approaches may result in an increased accuracy.

The characteristics of the OHCA data used in these studies may affect the sensitivity/specificity results, and in particular the characteristics of CPR, the selection criteria for VF, and the proportion of ASY among nonshockable rhythms. Rate and depth values of CPR in our data are similar to those reported in the original studies [21, 22] and represent the wide range of CPR characteristics found in the field. In particular, the CC rates are high (around 120 cpm), the spectral overlap with OHCA rhythms is therefore large, and suppressing the CPR artifact in our data should be challenging [8]. The CC depth was low even according to the 2000 resuscitation guidelines and lower than the 5 cm recommended in current guidelines [36]. However, no clear correlation between depth and larger artifacts has been demonstrated to date on human data. Our database only included VF annotated as coarse, as stated in the AHA statement. The three-phase model of cardiac arrest suggests that fine VF occurs when VF transitions from the electric phase into the circulatory or metabolic phases [37]. There is no conclusive evidence that immediate defibrillation is the optimal treatment in these latter phases of VF [38], so from a SAA design perspective it is a sound decision to only include coarse VF. On the other hand, our database has a large proportion of ASY among nonshockable rhythms (39%), in agreement with the fact that ASY is the most frequent nonshockable OHCA rhythm [39]. The high specificity of our method for ASY is particularly important because ASY is the most difficult nonshockable rhythm to detect during CPR [14, 16].

Our study shows that combining adaptive filtering with special SAAs that optimally diagnose the filtered ECG may result in an increased overall accuracy. In addition, the computational cost of the algorithm is low, as shown by the processing time analysis. The SAA algorithm computes at most six ECG features, and implementing our SVM in an AED requires only a few kilobytes of memory for the support vectors and the computation of the discriminant function (see equation (5)). The LMS algorithm using 5 harmonics involves only 10 coefficients [12], which substantially simplifies the filter. In any case, incorporating a CPR artifact filter to current AEDs is more complex than using algorithms that directly analyze the corrupt ECG [5, 6]. Filtering techniques based on the CD signal require the use of external CPR quality devices [40, 41] or modified defibrillation pads [42, 43] to record the acceleration signal. Alternatively other reference signals can be used, such as the thoracic impedance recorded through the defibrillation pads [19]. CPR artifact filters increase the complexity of the software and signal processing units of the AED and may even demand changes in its hardware to acquire reference signals.

Finally, several studies need to be completed before any method could be safely taken to the field. First, more conclusive results require testing the algorithm on data recorded by equipment different from those used for this study and with CPR delivered according to the latest 2010 CPR guidelines. In addition, retrospective studies based on complete resuscitation episodes should be conducted. In this way, the impact of using the method on CPR administration could be evaluated. This involves, among other things, a statistical evaluation of whether the method avoids unnecessary CPR interruptions in nonshockable rhythms and unnecessary CPR prolongations in shockable rhythms [36]. The methodology for such an evaluation has recently been developed [44].

## 5. Conclusions

This work introduces a new method for rhythm analysis during CPR with a novel design approach aimed at obtaining a high specificity. The method combines an adaptive LMS filter to suppress the CPR artifact with a new shock/no-shock classification method based on the analysis of the filtered ECG. The method resulted in an increased specificity of 96.6% without compromising the sensitivity, with overall performance figures that met AHA requirements.

## Figures and Tables

**Table 1:** Number of segments (patients in parenthesis) and characteristics of the CC rate and depth for the training and test datasets. Values for CC rate and depth are presented as median with 5–95 percentiles in parenthesis.

Rhythm type	Training			Testing		
	9-s seg.	Rate (cpm)	Depth (mm)	9-s seg.	Rate (cpm)	Depth (mm)
Shockable	**563 (35)**	**116 (92–143) **	**38 (25–47) **	**622 (34)**	**113 (89–157)**	**35 (20–50) **
Nonshockable	**3132 (110)**	**116 (88–155) **	**36 (20–51)**	**3350 (109)**	**116 (84–159)**	**35 (21–57) **
AS	1173 (66)	118 (92–164)	35 (18–52)	1309 (60)	117 (89–151)	34 (21–52)
ORG	1959 (66)	114 (86–149)	37 (23–51)	2041 (76)	116 (79–164)	35 (21–59)

Total	**3695 (123)**	**116 (89–151)**	**36 (21–51)**	**3972 (124)**	**116 (86–159) **	**35 (21–58) **

**Table 2:** Final classification for the 3-s windows and 9-s segments of the test set compared to the AHA performance goals. Sensitivities, specificities and low one-sided 90% CIs (in parenthesis) were obtained using GEE to adjust for clustering.

Rhythm type	3-s window		9-s segment		AHA goal [18]
	*n *	Se/Sp	*n *	Se/Sp	
Shockable	**1866**	**89.7 (85.5) **	**622 **	**91.0 (86.6)**	>90 (for VF)
Nonshockable	**10050**	**95.1 (94.3)**	**3350 **	**96.6 (95.9)**	>95
AS	3927	94.3 (93.1)	1309	96.5 (95.2)	>95
ORG	6123	95.6 (94.6)	2041	96.7 (95.8)	>95

**Table 3:** Comparative assessment in terms of accuracy and the composition of the databases (% of ASY in nonshockable rhythm in parenthesis) between the method proposed in this study and previous methods tested on OHCA rhythms.

Authors	Method	Accuracy		Testing datasets	
		Se (%)	Sp (%)	Sh	NSh
Eilevstjønn et al. [10]	MC-RAMP	96.7	79.9	92	174 (30%)
Aramendi et al. [19]	LMS filter	95.4	86.3	87	285 (31%)
Tan et al. [20]	ART filter	92.1	90.5	114	4155 (NA)
Li et al. [5]	Direct analysis	93.3	88.6	1256	964 (4%)
Krasteva et al. [6]	Direct analysis	90.1	86.1	172	721 (46%)
Proposed method	Filtering + SAA	91.0	96.6	622	3350 (39%)
